# Minos: variant adjudication and joint genotyping of cohorts of bacterial genomes

**DOI:** 10.1186/s13059-022-02714-x

**Published:** 2022-07-05

**Authors:** Martin Hunt, Brice Letcher, Kerri M. Malone, Giang Nguyen, Michael B. Hall, Rachel M. Colquhoun, Leandro Lima, Michael C. Schatz, Srividya Ramakrishnan, CRyPTIC consortium, Zamin Iqbal

**Affiliations:** 1 EMBL-EBI, Cambridge, UK; 2 Nuffield Department of Medicine, University of Oxford, Oxford, UK; 3 Institute of Evolutionary Biology, Ashworth Laboratories, University of Edinburgh, Edinburgh, UK; 4 Department of Computer Science, Johns Hopkins University, Baltimore, MD USA

## Abstract

There are many short-read variant-calling tools, with different strengths and weaknesses. We present a tool, Minos, which combines outputs from arbitrary variant callers, increasing recall without loss of precision. We benchmark on 62 samples from three bacterial species and an outbreak of 385 *Mycobacterium tuberculosis* samples. Minos also enables joint genotyping; we demonstrate on a large (*N*=13*k*) *M. tuberculosis* cohort, building a map of non-synonymous SNPs and indels in a region where all such variants are assumed to cause rifampicin resistance. We quantify the correlation with phenotypic resistance and then replicate in a second cohort (*N*=10*k*).

**Supplementary Information:**

The online version contains supplementary material available at (10.1186/s13059-022-02714-x).

## Background

The use of whole genome short-read sequence data to study cohorts of bacterial genomes from a single species is now a standard practice (e.g., [1, 2]). There are a multitude of variant callers, which analyze reads from a sample and make statements about where it differs from a fixed reference genome. However, there is no one best variant caller, or even approach—all have strengths and weaknesses. The single-sample-inference problem is well studied and understood—mapping to a reference works well where the sample and reference are reasonably close, and primarily for SNPs (SAMtools [3]), but fares progressively worse as the reference and sample diverge [4]. On the other hand, methods based on local assembly (GATK [5], Octopus [6]) are better able to detect small indels, and those based on global assembly (Cortex [7], McCortex [8]) are exceptionally specific, robust to reference-choice, and better at accessing clustered SNPs or indels up to a few kb in size, but at a cost in sensitivity. Ideally, it would be valuable to be able to combine the output of two different methods (“callsets”) in some rigorous manner, resulting in a product better than either. Simply using the union of callsets gives no control over false discovery rate, and the intersection is too conservative, losing the benefit of callers with different strengths. This “variant adjudication” problem of rigorously combining callsets, where discordances between input variants are resolved and then variant sites are genotyped, is the first challenge we address in this study. In doing so we separate two processes which are typically bound together within a single variant-caller: the discovery of genetic variants, and the genotyping of these variants. In our schema, we allow different callers to do discovery, and then use our new method to adjudicate (i.e., genotype).

Moving beyond single samples, there are many use-cases where one needs to jointly analyze a cohort, producing a matrix of variants versus samples and making binary or probabilistic statements (genotype calls) at all positions that are segregating in the cohort. This is more tricky, primarily because the density of variation increases with cohort size, and inevitably there are situations where SNPs and indels overlap. This is typically described as joint genotyping [5], and itself is a form of adjudication problem—once we have the full list of segregating sites and alleles, we can revisit all samples and genotype each one. This is the second main problem we address in this study.

**Fig. 1:**
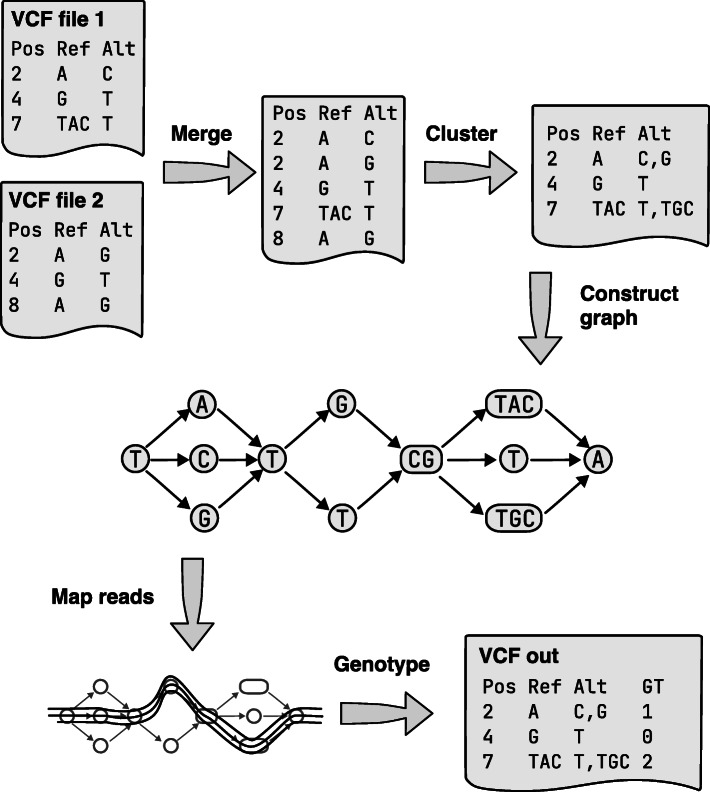
Variant adjudication pipeline implemented by Minos. Input variants in one or more VCF file(s) are merged to make a deduplicated set of variants. When running on a single sample, the input VCF files could be from different tools. When joint genotyping across samples, there is one VCF file originating from each sample. Next, overlapping variants are clustered together—for example the variants at positions 7 and 8—allowing the construction of a non-nested variation graph. Genotype calls are made using read mapping to the graph

Finally, underlying both, there is an important technical challenge: how to combine Variant Call Format (VCF) [9] files, either for the same sample from different variant callers, or from multiple samples in a cohort when collecting a list of segregating sites. In both cases, we want a clean VCF file with a set of non-overlapping records, each representing a segregating site with alternate alleles, which can be independently genotyped (see Fig. 1). This likely entails combining independent overlapping variants from the input VCF files into some consistent multi-allelic record. This particular problem is technically awkward and can in theory get arbitrarily ugly—in the pathological worst case, there could be overlapping records that gradually tile across the whole genome.

Our motivation was the desire to study tens of thousands of *Mycobacterium tuberculosis* genomes for the CRyPTIC project [10]. This project sequenced and phenotyped over 15,000 isolates for resistance to 13 different drugs using a 96-well microtitre plate [11], and then applied methods such as Genome-Wide Association Studies (GWAS) to analyze the genetic basis for drug resistance. *M. tuberculosis* has relatively low levels of diversity by bacterial standards, with no recombination, relatively few mobile genetic elements, and a small pan-genome [12]. However, despite this, almost 17% of the 4.4Mb genome was variable within the cohort, and in some regions almost every single base harbored a multiallelic SNP or indel. Typical approaches tuned for high precision callsets would refuse to make calls in such dense regions, but for our purposes we needed to be able to both represent and correctly genotype these regions.

There has been prior work on these problems, with recent tools focussed on human data. Joint genotyping is available in GATK; however, it relies on machine-learning-based filtering (VQSR) generated from human-specific truth-data. Applying GATK to non-human species required considerable efforts to train a black box VQSR for each new species (e.g., see [13] for *Plasmodium*). At the time of writing, GATK explicitly does not support bacterial data, although a new version for this is in development. There are also graph-based genotypers for structural variants that operate on similar principles to ours, although with different graph structures and genotyping models, such as Paragraph [14] and vg [15].

Two other graph-mapping based tools are available: BayesTyper [16] maps reads to a directed acyclic graph of informative kmers, and GraphTyper [17] maps to local graphs of SNPs and indels from pre-mapped reads. BayesTyper is set up to take VCFs from different callers, combine them and genotype, much like Minos. GraphTyper’s intended use is to genotype large human cohorts, either with SNP/indels it has discovered, or using a predefined VCF of structural variants. We compare Minos with both below.

Our approach was to build a pure adjudicator, able to run a single command that can take multiple VCF files, handle all overlapping variants, and output a single accurate callset with no inconsistencies. Intuitively, read pileup can be used to test goodness-of-fit. Reads should map perfectly to a reference containing the correct allele, so comparing pileups on alternate alleles can resolve disagreements between callsets. Of course, it would be prohibitively expensive to remap all reads to every input allele independently and then compare the pileup on each allele. Instead, we build a genome graph of the combined alleles from all callers and map once to that, using gramtools [18]. Reads naturally align to the correct allele, and we can genotype using the resulting coverage and ambiguity information (described below). We implemented this, plus a workflow for joint genotyping cohorts, in our new tool, Minos.

We first use 62 high-quality polished long-read assemblies from three bacterial species and benchmark Minos against BayesTyper and GraphTyper. We then apply all three tools to an *M. tuberculosis* outbreak (*N*=385) in the UK in 2013, evaluating precision for both reference and non-reference calls.

Finally, we apply our method to our motivating problem—studying antimicrobial resistance in large cohorts of *M. tuberculosis*. We first joint-genotype the CRyPTIC global cohort of *M. tuberculosis* genomes (*N*=15,215) which contains around 700,000 variants (roughly one SNP every 5bp). We focus on the 81bp rifampicin-resistance determining region (RRDR) in the *rpoB* gene, which bears an enormous level of variation. The WHO-endorsed Xpert^®^ MTB/RIF assay assumes any non-synonymous SNP or indel in this region causes resistance to rifampicin [19], although as we discuss below, the story is a little more complex. We restrict to non-synonymous SNPs and indels and give an unprecedented map of dense variation in the RRDR and how strongly each variant correlates with resistance. We find the five known “borderline” mutations [20] but also show that there are more. We then joint-genotype a second, independent, cohort of 13,411 *M. tuberculosis* genomes which have also been phenotyped for rifampicin, and replicate the finding. We consider the significance of these findings in the Discussion.

## Results

We developed a new tool called Minos, which takes putative variant calls as input, adjudicates between all of the calls, and reports a final accurate callset. It uses the standard Variant Call Format (VCF) for its input and output. It can accept VCF files from any source, using all records where the genotype (GT) field is present and has a non-reference call (records without this field are ignored). Additionally, Minos includes a Nextflow [21] pipeline to joint genotype large numbers (tens of thousands) of samples, producing a set of calls at the same variant sites across all samples. See Fig. 1 for an overview of the pipeline, and the “Methods” section for a complete description.

Existing tools to assess the accuracy of call sets, such as hap.py (https://github.com/Illumina/hap.py) and RTG vcfeval [22], were developed for human diploid data and require truth variant calls in a VCF file. Such evaluations typically need to cope with uncertain phasing in the “truth data”. However in our case, as is typical in bacterial genomics, the truth data is a polished (haploid) whole genome assembly assumed to contain no errors. We therefore developed a tool called Varifier to meet the need for a tool that uses such a truth sequence to determine the precision and recall of a call set. As described in full in the “Methods” section, Varifier evaluates each allele call by aligning the allele plus flanking sequence to the truth genome. This method is robust to complex variants, which can have more than one correct VCF representation. We found other tools could make errors around these types of variants—an example is given in Additional file 1. In this study, for each sample, we used a truth genome assembled from long reads (PacBio or Oxford Nanopore), and polished using the same Illumina reads that were used for variant calling. We used Varifier and these truth genomes to benchmark Minos against GraphTyper and BayesTyper with simulated and real data.

### Single-sample benchmarking

We performed an initial sanity check that BayesTyper, GraphTyper, and Minos all work as expected, using simulated reads made with ART [23] and simulated variants in the *M. tuberculosis* H37Rv reference genome [24]. All tools performed near perfectly on this simple data set, affirming that they make no major errors (Additional file 1: Fig. S1, Additional files 2, 3, 4 and 5: Tables S1–S4). The default Minos filters dropped the recall of SNPs and short indels slightly (Additional file 1: Fig. S1), due to unrealistically low variation in read depth in the simulations, which caused the “MIN_GCP” filter (see the “Methods” section) to fail true positive calls. However, as shown later, this is not an issue in real sequencing read data. Having confirmed all tools passed a basic test, we move on from the simulations to empirical data.

Next, the tools were compared using real data from *M. tuberculosis*, *Staphylococcus aureus*, and *Klebsiella pneumoniae*, using samples which each had a high-quality polished long read assembly to act as truth, and matched Illumina data (see Methods). We selected reference genomes for each species (1 for *M. tuberculosis*, 2 for *S. aureus* and 5 for *K. pneumoniae*) to reflect the diversity of the species.

For each Illumina data set, reads were trimmed using Trimmomatic [25], mapped to the reference genome with BWA MEM [26], and PCR duplicate reads were removed with SAMtools. Variants were called independently using two variant callers with orthogonal strengths: SAMtools/BCFtools is pileup-based, with high sensitivity for SNPs and low precision for indels; Cortex is assembly-based, with high precision for SNPs and indels, but lower recall. The SAMtools/BCFtools and Cortex callsets were input to BayesTyper, GraphTyper, and Minos, resulting in a single set of calls from each tool (BayesTyper and GraphTyper required additional processing of the SAMtools/Cortex VCF files, described in Additional file 1). In order to maximize recall, and because the adjudication tools should remove false-positive variants, the unfiltered callsets from SAMtools and Cortex were used. All results shown are using the default variant call filters for each tool, except where noted, and with unreliable regions of the genomes masked.

Note that GraphTyper can be run in two genotyping modes: default and “sv” (described in [27]). The “sv” mode resulted in significantly worse results in many cases (Additional file 6: Table S5); therefore, it is not discussed further in this manuscript. All GraphTyper results in this manuscript refer to running in default mode.

The results are summarized in Table 1 (also Additional file 1: Fig. S2, S3). Minos achieved the best F-score and recall across seven of the eight data sets. The biggest variation between tools was seen in the recall. Although GraphTyper had the highest precision, Minos was equally precise in three of the data sets, and the biggest difference in mean precision between Minos and GraphTyper was 0.05%.

**Table 1:** Mean precision, recall, and F-score on each empirical data set with each reference genome. Numbers in bold show the best precision, recall, and F-score for each species and reference genome

Species	Number of samples	Reference genome	Tool	Mean precision	Mean recall	Mean F-score
*M. tuberculosis*	17	H37Rv	BayesTyper	0.9995	**0.9217**	**0.9579**
			GraphTyper	**0.9997**	0.8938	0.9422
			Minos	**0.9997**	0.9181	0.9559
*S. aureus*	28	TW20	BayesTyper	0.9984	0.8669	0.9279
			GraphTyper	**0.9990**	0.7530	0.8545
			Minos	0.9988	**0.8786**	**0.9347**
		USA300	BayesTyper	0.9993	0.8671	0.9283
			GraphTyper	**0.9995**	0.7506	0.8534
			Minos	0.9994	**0.8792**	**0.9353**
*K. pneumoniae*	17	GCF_000784945.1	BayesTyper	0.9990	0.9052	0.9495
			GraphTyper	**0.9999**	0.9063	0.9505
			Minos	**0.9999**	**0.9143**	**0.9550**
		GCF_001952915.1	BayesTyper	0.9995	0.8800	0.9346
			GraphTyper	**0.9999**	0.8788	0.9340
			Minos	0.9994	**0.8922**	**0.9417**
		GCF_003073315.1	BayesTyper	0.9996	0.9267	0.9617
			GraphTyper	**0.9999**	0.9297	0.9634
			Minos	0.9998	**0.9367**	**0.9672**
		GCF_003076555.1	BayesTyper	0.9994	0.9397	0.9686
			GraphTyper	**0.9999**	0.9387	0.9683
			Minos	**0.9999**	**0.9438**	**0.9710**
		GCF_011006575.1	BayesTyper	0.9995	0.9078	0.9511
			GraphTyper	**0.9999**	0.9075	0.9513
			Minos	0.9998	**0.9238**	**0.9602**

We also investigated the effect of including more variant callers as input to BayesTyper, GraphTyper, and Minos. In principle, a new caller adds value if it finds variants which are missed by the other callers. The above analysis was repeated, but with calls from Snippy (https://github.com/tseemann/snippy) included together with those from SAMtools and Cortex. The results were almost identical: the mean precision and recall across all data for each tool was the same to two decimal places (Additional file 7: Table S6, Additional file 1: Fig. S4), indicating that for these data, Snippy offers no additional benefit once Samtools and Cortex are combined.

### Performance

On the real bacterial data, all tools had a relatively fast run time and low RAM usage (Additional file 8: Table S7, Additional file 1: Fig. S5). On each data set, GraphTyper had the shortest run time and smallest RAM usage, followed by Minos and then BayesTyper. The median wall clock time for Minos across the data sets ranged from approximately 2.5 min per sample (*M. tuberculosis*) to 6 min (*K. pneumoniae*). *K. pneumoniae* required the highest RAM, where the median peak usage across all runs for Minos was 2.5GB.

### Joint genotyping of cohorts

We wrote a Nextflow pipeline, included in the Minos code repository, to easily joint genotype large numbers of samples. It outputs a single VCF file containing all samples genotyped at the same sites, and the same information in per-sample VCF files. We tested this pipeline, together with BayesTyper and GraphTyper, to re-analyze 385 *M. tuberculosis* [28] samples from an outbreak in the UK, which we call the “Walker 2013” data set. Note that BayesTyper and GraphTyper are not set up for this use case, and therefore, processing the data was required to attempt to use these tools in this manner, as described in the Methods section.

Our final analysis was to joint genotype two large *M. tuberculosis* cohorts of more than ten thousand samples each, using the results to analyze the phenotypes and corresponding genotypes in the 81bp rifampicin resistance determining region (RRDR) of the *rpoB* gene. The first large data set (“CRyPTIC”) consisted of 15,215 samples released by the CRyPTIC project [10], phenotyped using a microtitre plate assay [11, 29], and the second (“Mykrobe”) data set consisted of 13,411 samples previously published [30] and phenotyped using traditional culture-based DST (drug susceptibility testing).

Variants were called on each sample independently with the same methods as above using Cortex and SAMtools, and then calls adjudicated with Minos. Each of these per-sample Minos VCF files was used as input to each of the three tools. The result was a callset for each tool, at the same variant sites for all samples for that tool (variant sites were not the same between tools). Minos is the only tool of the three that is set up to consistently merge all input VCF records, so that no two sites in the output contain reference positions in common. Furthermore, this means Minos does not, unlike BayesTyper and GraphTyper, output two separate VCF records with incompatible genotype calls. This is discussed in more detail in Additional file 1.

A summary of the data sets and variants output by Minos is given in Table 2. A typical *M. tuberculosis* genome might be around 1000 to 2000 SNPs distant from the H37Rv reference genome. Since these are spread across a 4.4Mb genome, excessive density of variants is not a problem for the single-sample adjudication problem. However when joint genotyping the larger cohorts, we are adjudicating all segregating variation, covering 17–18% of the genome, including some regions of hundreds of base-pairs where almost every single base is a multiallelic SNP or indel. These dense regions present a scaling challenge to graph genome algorithms, depending on implementation and indexing strategy.

**Table 2:** Summary of *M. tuberculosis* data sets used for joint genotyping. “Genome inside sites” is the total length of all reference alleles across all sites after clustering. It is reported as the total number of base pairs, and in parentheses as a percentage of the 4.4Mbp H37Rv reference genome. SNP sites is the number of sites where all alleles have length 1

Data set	Number of samples	Unique variants	Excluded variants	Sites after clustering	Genome inside sites (bp(%))	Total alleles	SNP sites
Walker 2013	385	31,548	231	30,621	41,437 (1%)	62,690	27,639
Mykrobe	13,411	699,484	6,259	593,584	756,003 (17%)	1,414,723	552,543
CRyPTIC	15,215	718,863	6,576	611,269	778,949 (18%)	1,469,100	568,224

### Outbreak analysis

We evaluated performance of Minos and alternate tools on genomes in the Walker 2013 data set, from an outbreak of *M. tuberculosis*. To measure the precision and recall of the three tools before and after joint genotyping, the 17 *M. tuberculosis* samples used earlier were added into the outbreak data set. Joint genotyping the samples generally had a negligible effect on precision and recall (Additional file 9: Table S8). BayesTyper recall increased by 0.07%, whereas the recall of GraphTyper and Minos dropped by 4.2% and 0.6% respectively. BayesTyper precision fell by 0.13%, and both GraphTyper and Minos increased by 0.01%.

**Fig. 2:**
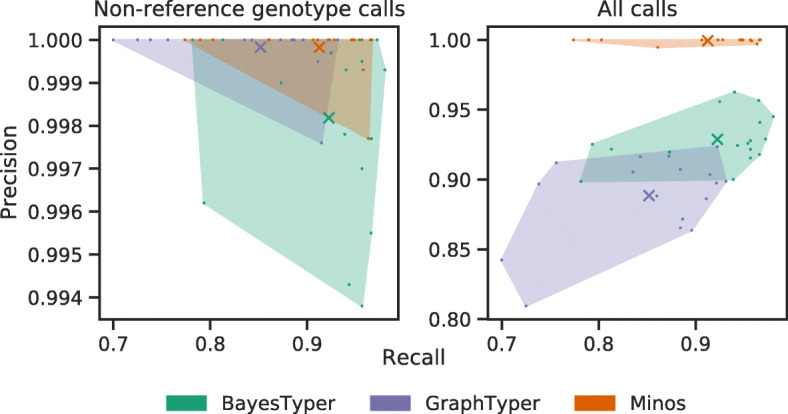
Precision and recall when joint genotyping *M. tuberculosis* outbreak data. The left plot considers non-reference allele calls only, i.e., the variant sites that are genotyped to be different from the reference genome. The right plot shows the results when all allele calls are included. Individual samples are marked as dots, and the mean precision and recall for each tool is shown as a cross. The convex hull of the data points for each caller is shaded with an associated color

Up to this point in the manuscript, precision has always been calculated by considering only non-reference allele calls, focussing on the differences between a given sample and the reference genome. However, joint genotyping involves genotyping every sample at every variant site. As a result, the majority of calls have the reference genotype, and correctly genotyping these cases is critical for applications such as building a phylogenetic tree, computing a genetic distance matrix or for GWAS. The difference between excluding or including reference calls for each tool is shown in Fig. 2 and Additional file 9: Table S8. After joint genotyping, and including reference genotype calls in the calculation, Minos achieved a precision of 99.95%, compared with 92.87% and 88.85% for BayesTyper and GraphTyper respectively.

### Association of rifampicin resistance with the RRDR region of *rpoB*

Returning to the motivating problem for which Minos was developed, we applied Minos to two large *M. tuberculosis* cohorts (“CRyPTIC” and “Mykrobe” data sets) for which we have associated resistance phenotype data for the first-line drug rifampicin. We sought first to confirm that Minos would indeed function at this scale, and then to build a detailed map of variation in the RRDR (rifampicin resistance determining region) of the *rpoB* gene. Minos output genotype calls at 593,584 and 611,269 variant sites for the Mykrobe and CRyPTIC data sets respectively (Table 2). These sites cover approximately 17–18% of the 4.4Mb H37Rv reference genome.

The total turnaround time of the pipeline, which was limited to a maximum of 2000 concurrent tasks, was approximately 23 h for the Mykrobe data set and 26 h for CRyPTIC. These represent real-world times, since the compute cluster we used was shared with numerous other users. A breakdown of the run times and maximum RAM usage is provided in Additional file 10: Table S9. The total CPU time was 787 core-days for the Mykrobe data set and 905 core-days for CRyPTIC. The pipeline is optimized to minimize memory, with the peak memory used when merging and clustering variants at less than 8GB. The majority of the run time comprises running Minos on each sample, which required less than 2GB of RAM per sample.

**Fig. 3:**
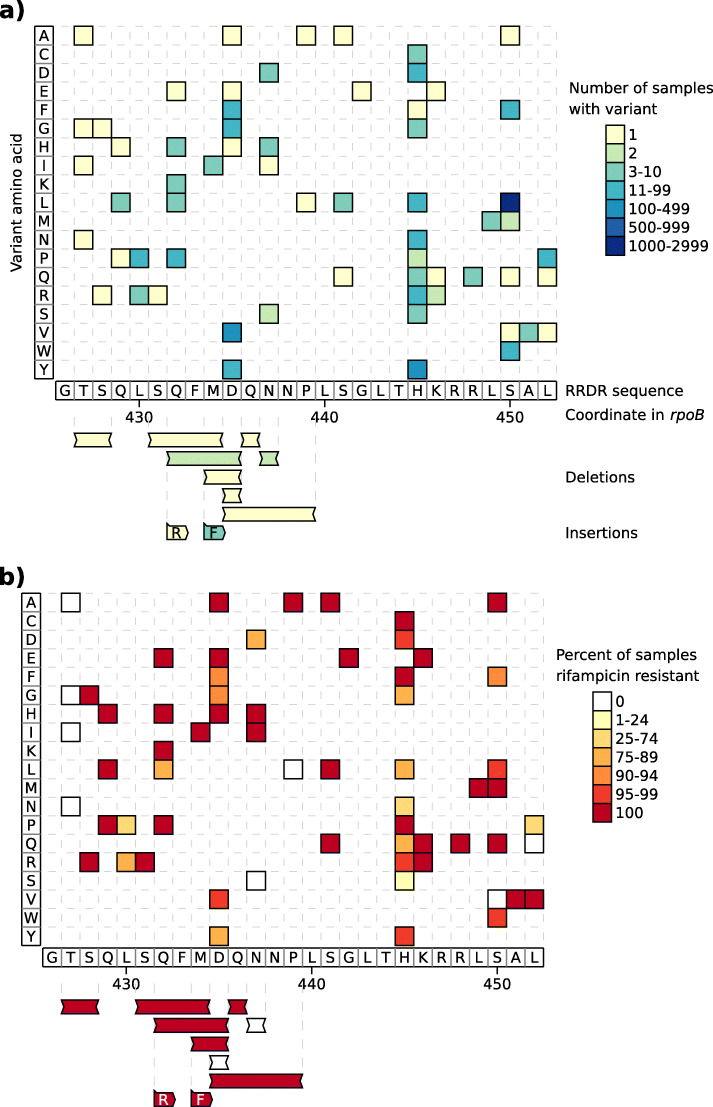
All amino acid variants identified in the RRDR of the *rpoB* gene by joint genotyping 8,955 samples from the CRyPTIC *M. tuberculosis* data set. Each plot shows the RRDR region from left to right. Single amino acid variants are shown in the upper grid, with the y axis corresponding to the variant amino acid. The lower area shows deletions and insertions, with the inserted sequence given in the colored boxes. For example, the leftmost deletion of amino acids TS at position 427-428 is found in one sample, which is resistant. The leftmost insertion adds R after the S at position 431 (found in one resistant sample). The plots show the same variants, but with different color schemes. In **a** each variant is colored by the number of samples possessing that variant. Plot **b** colors the variants by the percent of samples with that variant that are rifampicin resistant

We then used the joint genotyping output to analyze the genotype-phenotype relationship within the RRDR of the *rpoB* gene, by restricting to the 13,259 samples of the Mykrobe data set, and the 8955 samples of the CRyPTIC data set with a high quality rifampicin phenotype [10] (from 12,099 CRyPTIC samples with any quality rifampicin phenotype). Figure 3 shows all the identified amino acid variants—substitutions, insertions, and deletions—plotted along the RRDR, with their prevalence and proportion of resistant samples for the CRyPTIC data. The same plots for the Mykrobe data and all CRyPTIC data are given in Additional file 1: Fig. S6, and the raw data are in Additional file 11: Table S10.

As expected the variant S450L, found in 1919 of the samples, dominates. The next most common variant D435V appears in 345 samples. Several rare indels were identified, most of which appear to cause rifampicin resistance. In the CRyPTIC samples, two insertions were identified (R inserted at 431–432 and F at 433–434), and all nine samples with either of these are rifampicin resistant. Six of the eight deletions arise only in resistant samples. In the Mykrobe data, there were seven deletions and three insertions, all of which were only found in resistant samples.

We find a total of 72 distinct amino acid mutations and indels within the 81bp RRDR in the CRyPTIC samples with high-quality phenotypes, including the 5 mutations classified as “borderline resistant” by the WHO [20] (H445L, H445N, D435Y, L452P, L430P). There are 13 variants in the CRyPTIC samples where the number of susceptible samples is greater than the number of resistant samples. The most common are among the borderlines listed above—L430P, where 45/67 samples are susceptible (and 9/19 susceptible in the Mykrobe data), and H445N, where 16/22 samples are susceptible (2/6 in Mykrobe samples). The remaining such variants are rare, each seen in up to five samples. Full counts can be seen in Additional file 1: Fig. S6. Even if the known borderline mutations are excluded, Fig. 3 (right panel) shows a large number of moderate to low frequency variants with a range of correlations with resistance. We discuss these results further below.

## Discussion

Variant analysis from short read sequence data is by now a mature field, and there are many tens of different tools for detecting SNPs and short indels [31]. Setting aside performance issues and focussing entirely on completeness and correctness of the inferred SNPs and indels, it is clear that there is no single best tool. The relative weight given to mapping, assembly, paired-end information, and species-specific optimization (eg via machine learning) results in different strengths and weaknesses. This leads to two rational choices: first, to benchmark for your chosen application and choose the best tool, and second to find a way to combine the strengths of different callers. When setting up the CRyPTIC project, we observed that there was no off-the-shelf solution to this problem, and set out to produce an easy-to-use tool that would do this in a rigorous manner. Minos was the result, which we incorporated into a workflow for analysis of *M. tuberculosis* genomes called Clockwork. We found that in terms of single genome analysis, performance was relatively similar to other benchmarked tools—Minos generally had the best recall, and GraphTyper had marginally the best precision. However, only Minos would out of the box ingest two (or more) VCF files and output results; the other tools forced users to write code to prepare input data and glue together their processing stages. Combining VCF files with SNPs and indels is particularly challenging for cohorts, where a large proportion of the genome can be variable (17% in our CRyPTIC cohort for example). When analyzing the Walker 2013 *M. tuberculosis* outbreak and including reference/wild-type calls, Minos had much higher precision (7–10% higher) than the other tools, and on scaling up, only Minos could process the 12k and 13k CRyPTIC and Mykrobe cohorts.

Rifampicin is a bactericidal drug which is a critical component of the antitubercular arsenal, resistance to which is typically used as an epidemiological proxy for multi-drug resistance (defined as having resistance to both rifampicin and isoniazid), particularly in PCR-based rapid diagnostics such as the Xpert^®^ assay. The latest WHO technical report [20] showed that there were 6 known borderline mutations in *rpoB* (of which 5 were in the RRDR) [20, 32–34], and carefully reported the MIC (minimal inhibitory concentration) distributions for isolates with these mutations. In essence, the distribution of MICs (“the level of resistance”) overlapped with the distribution for wild-type (susceptible) *M. tuberculosis*, which leads to poor reproducibility of binary classification when the threshold between resistant and susceptible (ECOFF) lies in that overlap. Nevertheless, in the light of various reports of worse patient outcome associated with some of these mutations [35–40], the WHO expert group decided that all non-synonymous mutations and indels, even previously unseen ones, should be treated as causing resistance for the purposes of diagnostics and therapy. Our analysis of this, the largest consistently phenotyped cohort to date [10], reveals that the level of resistance caused by mutations in the RRDR is indeed heterogeneous, and reveals further candidate borderline mutations and indels, including a cascade of overlapping rare indels at position 431. This general picture is replicated in the second cohort (Mykrobe data set). For a more nuanced analysis, it is necessary to look at the MIC distribution associated with specific mutations (rather than using a binary resistant/susceptible classification), which has been done in [41].

The main limitation to this study is that for joint genotyping we set a deletion length limit of 50bp. This reflects an underlying design decision: Minos is a tool for combining VCF files from different callers or samples, adjudicating, and outputting an improved VCF file. However VCF is really not an appropriate file format for handling many overlapping small variants and large indels—for example, a 5-kb deletion covering 20 SNPs. We address this question of how best to genotype and encode multiscale variation (such as SNPs on top of long alternate haplotypes or SNPs under deletions) in a separate study [18].

## Conclusions

We have presented a new tool, Minos, that enables users to combine results from their preferred variant callers, integrating their strengths, to reach closer to the underlying truth. It also provides a method for joint genotyping SNPs and indels in bacterial genomes. As genomic analysis is now ubiquitous in bacteriology, we believe Minos will be of wide utility.

## Methods

### Minos pipeline

First we describe the methods used by Minos, which is implemented in Python and available under the MIT license at https://github.com/iqbal-lab-org/minos. The pipeline is outlined in Fig. 1. The first two stages process the input variant calls, which must be in one or more VCF files, to produce a single set of calls that can be used to generate a reference graph for read mapping. Initially, calls are normalized and deduplicated to make a single “merged” set of calls. These calls are then “clustered” into variant sites that define the variant graph used for read mapping. The merging and clustering are described below.

#### VCF merging

Each VCF file is processed individually as follows. Variant alleles to be retained for further processing are extracted, where for each record if the genotype (GT) field is present then only called the alleles are kept; otherwise, all alleles are used. The remaining records and their alleles are written to a new VCF file. Variants are decomposed into unique SNPs and indels using the commands vcfbreakmulti and vcfallelicprimitives -l 10000 from vcflib (https://github.com/vcflib/vcflib), followed by the normalize function from vt [42], and finally the vcfuniq command from vcflib. These VCF files are loaded into a single data structure containing all the unique variants, plus the origin (i.e., which VCF file) of each variant.

#### Variant clustering

The merged variants are used to produce a single VCF file of “clustered” variants that is compatible with gramtools, which in turn is used to generate a variant graph and map reads to that graph. Although gramtools supports more complex situations (SNPs on alternate haplotypes separate from the reference genome, SNPs underneath long deletions) [18], we restrict to “non-nested” variation to maintain compatibility with VCF. Therefore, overlapping variants must be converted into a single variant site (i.e., line in a VCF file) containing multiple alleles (an example is given in Additional file 1: Fig. S7). This is straightforward when processing a single sample with a few input VCF files, such as the SAMtools and Cortex VCF files used in this study when benchmarking Minos against other tools. Where possible, all combinations of alleles are generated and included in the graph. However, this is not always feasible when genotyping a large number of samples (hundreds or thousands) because the number of theoretically possible alleles at one site could be very large.

To process a large number of samples, heuristics are used to simplify the variant graph. First, the number of alleles is limited by only allowing deletions of length (by default) ≤50bp. This prevents a combinatorial explosion where SNPs underneath the deletion can cause impractically large numbers of alleles: *n* biallelic SNPs generates 2^*n*^ alleles. Second, the number of alleles in a variant site is limited to (by default) 500. If generating all allele combinations at a site results in too many alleles, then only combinations of alleles actually seen in each sample are used. This happened at 1252 sites in the Mykrobe data set, covering 53,632bp of the reference genome, and at 1195 sites (covering 50,324bp) in the CRyPTIC data set. Third, it is possible for the graph to contain the same sequence more than once across multiple variant sites, by choosing different paths through the graph (an example is given in Additional file 1: Fig. S8—roughly this can happen in low complexity sequence where two alternate deletions of some repetitive sequence can lead to the same final sequence). As each new variant site is added, the previous seven sites are checked and any sites generating duplicate sequences are merged into a single deduplicated site. Removing these duplications is necessary to prevent downstream read mapping issues caused by ambiguous mapping to different paths in the graph that are really the same sequence. Finally, to reduce RAM usage and run time, there is an option to split the graph into chunks, with read mapping run separately on each of these chunks. Using this option requires the reads to be in a sorted indexed BAM file, so that the reads for each chunk can be efficiently extracted for mapping.

#### Graph mapping and genotyping

The clustered VCF file made in the previous stage is input to the build command of gramtools [18] to make a variant graph for read mapping. Reads are mapped to the graph using the gramtools command quasimap. Each variant site is genotyped using the output from gramtools, which reports the number of reads mapped to each allele, and the read depth across each position of each allele. Minos supports haploid genotyping calling only, using the model described below. Minos and gramtools use similar models—the gramtools model was based on that of Minos, but was modified to handle nested genotyping.

At each variant site, the aim is to choose the correct, i.e., most likely, allele from a set of alleles *A*. We have the following information from gramtools:

1. A function $\gamma :P(A)\rightarrow \mathbb {N}$ (where *P*(*A*) is the power set of *A*), defined by *γ*(*X*)= the number of reads that map to all alleles in *X* (and map to no other alleles). Since gramtools only reports the combinations of alleles that it sees, we define *γ* by assuming that all elements of *P*(*A*) not reported by gramtools have zero reads. Note also that gramtools does exact matching of the full read length only - clipping the ends of the reads or mismatches between the read and graph are not allowed;
2. For each allele *a* belonging to *A*, the read depth at each position in *a*, where reads are allowed to be multiply mapped. This means that for each allele gramtools matches a read to, a per-base coverage counter of each allele’s matching bases is incremented. Thus, if a read maps to the middle 100bp of two long alternative alleles, then a counter is incremented at each position of those 100bp in each allele.

Let *c* be the total coverage at a site, given by

$$ c = \sum_{X\in P(A)} \gamma(X).$$

Let *a* be an allele belonging to *A*. Define the coverage *c*_*a*_ of *a* to be

$$ c_{a} = \sum_{X\in P(A) : a \in X} \gamma(X).$$

Let *ε* be the error rate in the reads, for which a default value of 0.002 is used and can be changed by the user. Let *d* be the expected read depth and *σ*^2^ the read depth variance, which are estimated using the read depth reported by gramtools at each variant site. We assume that the read depth follows a negative binomial distribution NB(*n*,*r*), where the parameters *n* and *r* are given by

$$ n = \frac{d^{2}}{\sigma^{2} - d} \quad \textrm{ and} \quad r = \frac{\sigma^{2} - d}{\sigma^{2}}.$$

This requires *σ*^2^>*d*. If this is not the case, then we set *σ*^2^ to be double the read depth *d*. This is only expected to happen in rare circumstances and was only seen in the simulated data sets where the read depth was very even, unlike real data. The genotyping model used by Minos comprises the three terms:

1. “Correct” coverage: NB(*n*,*r*,*c*_*a*_);
2. Coverage due to read errors: $\phantom {\dot {i}\!}\varepsilon ^{c-c_{a}}$;
3. A gap (i.e., zero coverage) penalty: $p^{\frac {b}{\ell }} (1-p)^{\frac {\ell -b}{\ell }}$, where *p*=1−NB(*n*,*r*,0) is the probability that a given position has zero depth, *ℓ* is the length of allele *a*, and *b* is the number of positions in *a* with non zero coverage.

The log likelihood is then calculated by summing the natural logarithm of these three terms. The allele with the greatest log likelihood is chosen, with genotype confidence of the difference in log likelihoods of that allele and the second greatest log likelihood. The genotype confidence is reported in the Minos output VCF file using the tag GT_CONF.

#### Variant call filtering

The FILTER column of the VCF file made by Minos is implemented using four filters. The first requires a read depth of at least two, called MIN_DP in the output VCF file. The second is a read depth no more than the mean depth plus three standard deviations, called MAX_DP. The third filter identifies apparent heterozygous calls (for example, caused by contamination), requiring by default at least 90% of the reads to support the called allele. It is called MIN_FRS (“minimum fraction of read support”, can be set by the user). The final filter removes low confidence genotype calls. Since the genotyping model is dependent on read depth, the confidence score is not directly comparable between different sets of reads. This is accounted for by normalising the confidence score as follows. 10,000 SNPs are simulated by sampling read depths from a negative binomial distribution, defined by the observed mean depth and variance—this is the same distribution as used in the genotyping model. Incorrect read depth is sampled from a binomial distribution with *n* = observed read depth, and *p* = read error rate. These simulated SNPs are genotyped using the same method as used when variant calling, generating an expected distribution of genotyping confidence scores specific to this run. When genotyping the real variant sites, any call with a confidence score in the first 0.5% of the simulated genotype score distribution fails the filter (the threshold can be set by the user). This filter is called MIN_GCP (minimum genotype confidence percentile) in the output VCF file.

#### Joint variant calling

The Minos Nextflow pipeline for joint genotyping large sets of samples is conceptually very similar to running on a per-sample basis and proceeds as follows. The starting point is a VCF file of variant calls for each sample. These VCF files are clustered and merged, as described above, to produce a single gramtools graph for variant calling all samples. This graph should encapsulate all variants found across all of the input VCF files (except for deletions longer than 50bp). Each sample is genotyped using its reads mapped to the gramtools graph, resulting in a VCF file for each sample, where the variant sites are identical across all samples. The pipeline also produces a single multi-sample VCF file, combining the files using ivcfmerge (https://github.com/iqbal-lab-org/ivcfmerge). Finally, a distance matrix is calculated by defining the distance between any two samples to be the number of variant sites where those samples have different genotype calls.

### Variant call evaluation

The variant call evaluation with Varifier was implemented in Python and is available under the MIT license at https://github.com/iqbal-lab-org/varifier. The required input is as follows: (1) a VCF file of variant calls to be evaluated; (2) a “mapping genome” FASTA file, which is the reference sequence corresponding to the VCF file; and (3) a “truth genome,” which is the sequence assumed to be correct. The basic idea is to assign a score from zero (meaning false-positive) to one (true-positive) to each variant call, where fractional scores indicate partially correct calls. The score is determined by mapping probe sequences generated from the reference and alternative alleles to the truth genome.

### Precision

Each variant is processed using the following method. First, variants with no genotype call (GT field) or a heterozygous genotype are ignored. This method is designed for haploid organisms only since it essentially looks for perfect allele matches to the truth genome, which does not work for heterozygous genotype calls. A probe sequence is generated comprising the called allele, plus the (by default) 100 flanking nucleotides from the mapping reference before and after the allele—we call this the “alt probe”. Similarly, a “ref probe” is generated that uses the reference allele instead of the called allele. The alt probe is mapped to the truth genome using minimap2 [43], and mappings that do not include the allele in the alignment (i.e., if the start and end positions of the allele in the probe do not lie completely inside the start and end positions of the mapping because of soft-clipping) or have mapping quality equal to zero are ignored. If there are no remaining mappings, then the variant is classified as a false positive and assigned a score of zero. Otherwise, the minimap2 mapping that has the greatest number of allele positions matching the mapping genome is chosen as the “best” match. Similarly, the ref probe is mapped to the mapping genome and the best mapping is chosen, but with the additional requirement that the alignment start position in the mapping genome must be equal to that of the best alt probe mapping. If this results in no ref probe mapping, but with the alt probe mapped, then the variant is classified as a true positive and assigned a score of 1.

If both probe sequences have a best match identified, then edit distances between sequences are used to define a score for the variant call. For motivation, consider the following relatively simple example allele sequences:


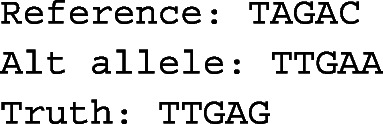


Although the called allele is incorrect because it missed the C to G SNP, it does include the other A to T SNP and is 80% correct (4/5 of the sequence matches the truth). However, the called allele only contains half of the correct variation between the reference and the truth (1/2 SNPs are called), and we would like to account for this. To avoid long insertions or deletions dominating results, we score an insertion or deletion of any length as 1 (i.e., the same as a SNP) when calculating edit distance. Let *d*(*t*,*r*) be the edit distance between the truth and reference alleles, and *d*(*t*,*a*) the edit distance between the truth and alternative alleles. We define the score as zero if *d*(*t*,*r*) is zero, otherwise:

$$ 1-\frac{d(t,a)}{d(t,r)}. $$

In the example above, the score is 1−(1/2)=0.5. Note that in the simple case of a single SNP, a false-positive scores zero and a true positive scores one. This edit distance-based measure is designed to handle indels and other complex variants. Although the score is usually between zero and one inclusive, in rare cases where the called allele is very distant from the truth, it is possible to have a negative score. The overall precision is calculated by dividing the total of the numerators by the total of the denominators, summed over all variants under consideration.

### Recall

Recall is determined using the following method (a flow chart is provided in Additional file 1: Fig. S9). First, a VCF file of all expected calls must either be supplied by the user, or alternatively is made by comparing the mapping genome to the truth genome. Two separate expected callsets are made: one using MUMmer [44] and the other using minimap2 and PAFtools. MUMmer is used by running the commands from its dnadiff pipeline. minimap2 is run with the options -c –cs, and the output is piped into unix sort -k6,6 -k8,8n, and then into the call command of PAFtools with the options -l50 -L50. Taking the union gives a set of variant calls between the mapping and truth genomes, which we expect to contain false positives that need to be removed. The final truth variant callset is made using the same probe mapping method described in the above precision section, to remove false-positive calls. Each variant call from MUMmer and minimap2 is kept if probe mapping to the truth genome results in a true-positive call where the called allele matches perfectly (in other words, the variant has a score of one). In the rare case where both tools call at the same position but with conflicting calls, the calls are not used.

To recap, at this point in the recall pipeline, we have a set of variants to be evaluated, a set of truth variant calls, and the mapping and truth genomes. The truth calls are in a VCF file with respect to the mapping genome. Next, all variants in the VCF file of calls to evaluate are applied to the mapping genome, to produce a new “mutated” genome. Determining recall is now the same as answering: how many truth variants are found in the mutated genome? This is answered using probe mapping using the same method as for precision. The VCF file of truth calls is evaluated, where the mutated reference takes the place of the “truth” genome.

### Benchmarking

#### Truth genomes

The *S. aureus* truth genomes were generated from PacBio and Illumina reads as follows. The PacBio raw reads were assembled using canu v1.6 [45] to produce the draft assembly. The contigs from the draft assembly were aligned to the respective reference genomes using the nucmer utility from the MUMmer3 [46] package. The contigs were oriented to match the reference and trimmed based on the nucmer alignments and circularized using minimus2 [47]. The assemblies were polished using the Illumina reads by iteratively running Pilon v1.23 [48] until no more corections were made, up to a maximum of 10 runs. Reads were mapped using BWA MEM version 0.7.17 to make input for each Pilon iteration.

The 17 *M. tuberculosis* truth genomes were from [18], which already had the same Pilon polishing process applied to them as used on the *S. aureus* genomes. We used *K. pneumoniae* assemblies from [49] for the truth genomes, which we note already had Pilon run on them as part of their assembly process.

#### Genome masks

A genome mask was available for *M. tuberculosis* H37Rv, which is used routinely by Public Health England. The plasmids were masked from the *K. pneumoniae* and *S. aureus* mapping genomes. A mask was generated for each truth genome by excluding positions where there was not a majority agreement between Illumina mapped Illumina reads and that genome, as described in Additional file 1. These masks were used by Varifier, which ignores all variants intersecting any masked region of the truth or mapping genomes.

#### *K. pneumoniae* reference genomes

The five *K. pneumoniae* genomes used as references for variant calling were chosen as follows. The average nucleotide identity (ANI) was calculated between each truth genome and all *K. pneumoniae* genomes in RefSeq using FastANI [50]. RefSeq [51] genomes with a minimum ANI less than 97.5% or a maximum ANI of 100% were excluded. The remaining genomes were listed in order of the minimum ANI, and 5 the genomes were chosen evenly spaced from this list, to obtain a range of ANI between the truth and mapping genomes.

#### Joint genotyping

The nextflow pipeline included in the Minos github repository was used to run joint genotyping on all three data sets. A nextflow configuration file is included that contains preset profiles to set sensible default parameters for “medium” and “large” size data sets. The “medium” profile was used for the Walker 2013 data set, and “large” was used for the CRyPTIC and Mykrobe data. The pipeline needs an input TSV file, listing sample names and paths to VCF and reads files, plus the reference genome in FASTA format, and optionally a reference genome mask in BED format. The single command line used to run the complete pipeline is provided in Additional file 1. The pipeline is set up to handle large cohorts by saving RAM where possible. It splits the genome into slices, each containing approximately the same number of alleles, and processes each slice in series. Each slice overlaps by a read length to remove end effects from mapping. The Walker 2013 set was split into 100 slices, and the two large sets into 300 slices. The slicing option requires that the input reads for each sample are in a sorted, indexed BAM file, so that the reads for each slice can be efficiently extracted. Such BAM files are typically generated during most variant calling pipelines, and so this requirement is unlikely to create further work.

Joint genotyping with BayesTyper and GraphTyper both required a single sorted VCF file from concatenating (taking account of VCF headers) all input VCF files. This file was sorted, compressed with bgzip, and indexed with tabix for GraphTyper. For BayesTyper, bcftools norm was run on the VCF file. Then the same commands used for running on individual samples were used, as described in Additional file 1. These were successful on all samples in the Walker 2013 data set. On the Mykrobe data set both tools failed on the first sample, ERR025833. For BayesTyper, the combine function ran successfully, but the cluster command failed. GraphTyper failed when running the genotype function, after processing approximately 10% of the genome, with a peak RAM usage of 76GB.

After joint genotyping the CRyPTIC and Mykrobe data sets, the RRDR region was analyzed as follows. To avoid ambiguity, we note that the RRDR region is the 27 amino acid sequence at 426-452 in the *rpoB* gene in the H37Rv genome (it is often alternatively described with *E. coli* numbering as 507-533). In H37Rv genome coordinates, this is 759807-763325.

To analyze the RRDR region, only samples that had a high-quality rifampicin phenotype of resistant or susceptible were used. This information is provided in the supplementary tables of [30] for the Mykrobe data. For the CRyPTIC data, we used the samples from [10]. Each of these samples was processed as follows. Its variants contained in the whole *rpoB* gene were extracted and applied to the genome sequence and translated, making a mutated amino acid sequence. The amino acid variants of the sample were deduced from aligning the mutated amino acid sequence to the reference amino acid sequence. Then the variants in the RRDR were extracted for each sample. In this way, combinations of nucleotide variants were accounted for (for example, two consecutive SNPs could cause a single amino acid change).

## Supplementary Information

**Additional file 1** Supplementary text and supplementary Figures S1-S9.

**Additional file 2** Supplementary Table S1. Raw results generated using calls from Cortex and SAMtools.

**Additional file 3** Supplementary Table S2. Raw results generated using calls from Cortex, SAMtools and Snippy.

**Additional file 4** Supplementary Table S3. Results of calling simulated SNPs, insertions and deletions in the *M. tuberculosis* genome.

**Additional file 5** Supplementary Table S4. Results of calling simulated complex variants in the *M. tuberculosis* genome. Total length is the length of the ref allele. Columns 2–5 show the number of each type of variant added to make the complex variant. For example, the first set of variants was 3 SNPs in a window of 10bp, and no insertions or deletions. The second set was 3 SNPs, one insertion of 2bp, and one deletion of 2bp in a window of 10bp.

**Additional file 6** Supplementary Table S5. Results of all tools evaluated on the empirical bacteria data set. Results are using the default filter of each tool, which means only taking VCF records where the FILTER column was equal to PASS. The “unfiltered” results are from ignoring the FILTER column and using all records.

**Additional file 7** Supplementary Table S6. Summary of effect of including variants from Snippy, in addition to Cortex and SAMtools, as input to BayesTyper, GraphTyper, and Minos. The values are the mean change in precision and recall calculated across all simulated data and all bacteria data.

**Additional file 8** Supplementary Table S7. Run time and memory summary for each tool on each data set. Values are taken from the output of the Unix command time -v.

**Additional file 9** Supplementary Table S8. Change in accuracy before and after running the joint genotyping pipeline on the Walker 2013 *M. tuberculosis* data. Joint genotyped precision is calculated in two ways: using just the non-reference allele calls, and using all calls. Note that this does not apply to the recall because in that case we only look for non-reference calls, and so including reference calls has no effect.

**Additional file 10** Supplementary Table S9. Joint genotyping Minos memory usage and run times. Values were taken from the LSF reports for each Nextflow task (“Max Memory” and “Run time”). The first three stages VCF merge, VCF cluster, and Gramtools build used 10, 20 and 20 CPUs respectively. The run times are quoted here, not total CPU time. ^1^Minos is run on each sample in parallel. The mean run time per sample and maximum RAM across all samples is shown. ^2^This process in an intermediate stage used when producing the final merged VCF file. It merges batches of 300 VCF files each into one VCF file. The merged VCF files are then input to the last task VCF final merge. The mean run time for each batch and maximum RAM across all batches is shown.

**Additional file 11** Supplementary Table S10. Counts of resistant and susceptible phenotypes for each amino acid variant seen in the RRDR region of the *rpoB* gene for the CRyPTIC and Mykrobe data sets. Counts are shown for all CRyPTIC samples with a rifampicin phenotype (CRyPTIC all) and only for those with a high quality phenotype (CRyPTIC high qual). Asterisk (*) shows borderline resistant mutations identified by the WHO.

**Additional file 12** Supplementary Table S11. *M. tuberculosis* accessions.

**Additional file 13** Supplementary Table S12. *K. pneumoniae* accessions.

**Additional file 14** Supplementary Table S13. *S. aureus* accessions.

**Additional file 15** Review history.

## Data Availability

The reference genomes used for variant calling were as follows. • *M. tuberculosis*: H37Rv version 3 reference genome NC_000962.3 [24]. • *S. aureus*: USA300 genome GCA_000013465.1 [52] and the TW20 genome GCA_000027045.1 [53]. • *K. pneumoniae*: GCF_000784945 [54], GCF_001952915 [55], GCF_003073315, GCF_003076555 [56], and GCF_011006575 [57]. A data download is available from Figshare [58] that contains FASTA files of all truth genomes and BED files of all genome masks. Accessions for reads and assemblies are provided in Additional files 12, 13 and 14: Tables S11–S13 and are also included in the data download in tab-delimited format. The Mykrobe data set accessions are in the Supplementary file from [30], available at [59]. Details of the CRyPTIC data can be found in [10]. The pipeline to call variants, run BayesTyper, GraphTyper, and Minos, and run Varifier was written in Python and is available under the MIT license from Github [60]. A copy of the two singularity [61] containers used for this study are available from Figshare: [62] was used for all benchmarking except for [63], which was used for analysis that used Snippy calls. All software versions and command lines used are in Additional file 1.
