# Supplementary material for: Minos: variant adjudication and joint genotyping of cohorts of bacterial genomes
Source: Genome Biol. 2022 Jul 5;23:147. doi: 10.1186/s13059-022-02714-x (PMC9254434; doi:10.1186/s13059-022-02714-x)
Supplement: Supplementary file 15 — Additional file 15 Review history. [file 13059_2022_2714_MOESM15_ESM.docx]

Review History

**First round of review**

**Reviewer 1**

**Were you able to assess all statistics in the manuscript, including the appropriateness of statistical tests used?**

Yes, and I have assessed the statistics in my report.

**Were you able to directly test the methods?**

Yes.

**Comments to author:**

The authors present a new method, Minos, which combines variants from multiple variant callers and outputs a new set of variants. The authors call this process "variant adjudication". The aim of the tool is to create a final set of non-overlapping set of variants from all the input VCFs. The authors demonstrate some use cases for variant adjudication, in single sample calling it is possible to merge different call sets of the same sample into one, resolving the disagreements between different calling methods. The authors also demonstrate they can jointly call a bacteria cohort by constructing a graph containing the combined variant allele sequences, map reads to the graph, and then determine their genotype based on the graph mappings. This is opposed to tools such as GATK that use gVCFs for joint calling, however the authors claim GATK does not currently support bacterial data.

Overall the paper is well written and easy to follow. The results show that Minos is useful in practice and I want to thank the authors for their contribution and making the Minos code and benchmarks available. I have a few points though that I think should be addressed though:

* Based on the abstract and introduction, it seems that Minos should work with VCFs produced by any variant caller. However, I did not find it explicitly stated which callers are supported and throughout all benchmarks only two methods are tested (SAMtools and Cortex). Can you clarify why you solely stuck with those two methods? I.e. is Minos somehow limited to these two exact methods? Would the results not be better if you picked three or more input methods?

* According to the introduction, Minos addresses a problem with previous methods that they produce contradictory output. An example of the problem is discussed in Supplementary section 2 - where a deletion overlaps a SNP. A deletion call of "0" and SNP call of "1" is said to be contradictory/inconsistent, however I think this is a correct usage of the "*" allele in VCF. This output would mean that the SNP is present but the deletion is not. The format has each "variant event" (SNP/indel) in separate lines which is often preferred and is used by many other variant callers too, such as GATK and Octopus.

* The authors also provide a new tool for evaluating the output VCF files, Varifier. However, I found the motivation behind its need a bit unclear as there are already many good solutions available (RTG vcfeval, Illumina's Hap.py, syndip). What are the advantages of using Varifier over previous methods?

* I think there might be an error in Varifier's formula for calculating precision. For example, in the case of a simple false positive SNP (REF=A, ALT=T, TRUTH=A) then d(t,r)=0 and d(t,a)=1. This is supposed to get a score of 0 according to the text but with the given formula you get (0-1)/0 = -1/0

* Another concern with Varifier's precisions calculations is the use of edit distance since it means indels can weigh a lot more than SNPs. For example, a single 500 bp insertion false positive is equivalent to making 500 different SNP false positives. This means SNP precision has much lower effect on the final precision metric compared to other evaluation tools. Because of this, would you also consider stratifying somewhere the precision also by SNP and indel variants?

**Reviewer 2**

**Were you able to assess all statistics in the manuscript, including the appropriateness of statistical tests used?**

Yes, and I have assessed the statistics in my report.

**Comments to author:**

This paper describes a methodology implemented in a python software package called Minos. The goal of this method is to call variants or genotype individual samples w.r.t. reference cohort. The application focus is on bacterial genomes, where overlapping variants are more common.

The novelty of the method is to leave the discovery of variants to other established tools and focus on genotyping individual samples. The reads from bacterial species are fed through various variant calling programs and the variants recorded. Minos then takes the set of variants discovered and builds a variant graph from all programs and all samples. The reads for each samples are then aligned to the variant graph and the optimal variant is chosen as the output.

This approach, which the authors describe as "adjudicating" (I had too google that and double check the spelling), has the advantage of combining both output from several programs, each with their strengths and weaknesses, as well as sharing information across samples/species.

The introduction serves as a review of previous methods as well as motivating the problem. It is well written but I would urge the authors to make a clearer distinction between the problem of variant discovery (just finding the presence or absence of a variant) and genotyping (given the set of variants, which one does this individual have). Many programs treat this as a single problem, often termed as single sample variant calling in Human datasets, or separate them when doing joint variant calling.

In particular, pg2, lines 59-page3 line3, this comparison of tools that cannot merge VCF files for bacterial species, it is mentioned that they were developed for human data primarily, but Graphtyper was also developed to go from BAM to genotypes for tens of thousands of human genomes and merging conflicting vcf's was not a primary design requirement. Overlapping variants are however dealt with in the variant discovery phase of the problem.

Overall this is a well written paper that focuses on a problem of importance when genotyping bacterial samples. The use of long read datasets for benchmarks as a gold standard is very important, but in the discussion the authors might want to address why we would want to use short read data at all.

Comments:

On pg 5, line 6, you run Graphtyper only as a genotyper and not as a variant discovery tool, I'm pleasantly surprised to see that it works on bacterial genomes, but it would be interesting to see how well it does when run as a variant discovery tool.

Pg 6, table 1

The comparison is quite close in many of the cases between the three methods. All of them are adjudicating the set of discovered variants and genotyping each sample, but it would be interesting to compare it to a) single sample variant callers and b) joint genotyping tools, I think the addition of those comparison would make it clear how important this step is.

Pg 7, line 49

The 23 and 26 hours on 2000 concurrent tasks is hard to compare, I would recommend reporting the total number of CPU hours.

pg 8, line 38. "which appear to cause rifampicin resistance"

How is this evaluated?

pg 9, Figure 3.

Although it is cited that variations in the rpoB gene can cause rifampicin resistance, the only purpose of figure 3 is to show that the variants discovered with Minos are in many of the resistant species. Now there are two questions

1. How well did the other programs do in finding/calling these variants?

2. If you compare other genes, not associated with rifampicin resistance, do you see the same enrichment of variants

3. How much information can be extracted from this, e.g. is there a statistical associatioon that these variants are more likely to be present in resistance and absent in non-resistant species? Any way you can quantify would be good.

pg 11, line 25

"combinations of alleles actually seen is used"

How often does this happen in the datasets?

pg 11, line 57

"mismatches between reads and graph are not allowed"

is this global or local, does the entire read have to match?

pg 12, line 8

"at a site", (missing A)

pg 13, line 46

"mappings that do not include the allele in the alignment"

This could be reworded to make it clearer, does this mean if the read is softclipped or something else?

pg 14, line 12

1-\frac{d(t,a)}{d(t,r)}

would be clearer in my opinion

Installation:

I tried to clone the repo and install, but I failed too build gramtools. When I did it manually (clone and run cmake) it complained that it required openMP, which my default mac compiler doesn't support.

**Authors Response**

**Point-by-point responses to the reviewers’ comments:**

Reviewer #1:

The authors present a new method, Minos, which combines variants from multiple variant callers and outputs a new set of variants. The authors call this process "variant adjudication". The aim of the tool is to create a final set of non-overlapping set of variants from all the input VCFs. The authors demonstrate some use cases for variant adjudication, in single sample calling it is possible to merge different call sets of the same sample into one, resolving the disagreements between different calling methods. The authors also demonstrate they can jointly call a bacteria cohort by constructing a graph containing the combined variant allele sequences, map reads to the graph, and then determine their genotype based on the graph mappings. This is opposed to tools such as GATK that use gVCFs for joint calling, however the authors claim GATK does not currently support bacterial data.

Overall the paper is well written and easy to follow. The results show that Minos is useful in practice and I want to thank the authors for their contribution and making the Minos code and benchmarks available. I have a few points though that I think should be addressed though:

* Based on the abstract and introduction, it seems that Minos should work with VCFs produced by any variant caller. However, I did not find it explicitly stated which callers are supported and throughout all benchmarks only two methods are tested (SAMtools and Cortex). Can you clarify why you solely stuck with those two methods? I.e. is Minos somehow limited to these two exact methods?

*Reply: We apologise for the lack of clarity. As the reviewer surmises, Minos does indeed work with VCFs produced by any variant caller. We now explicitly state this:*

*“It can accept VCF files from any source, using all records where the genotype (GT) field is present and has a non-reference call (records without this field are ignored).”*

*The core functionality of minos is to combine two different VCFs for a single sample, and produce a single VCF which incorporates all of the calls, choosing the correct call where there is any conflict between the inputs. There are two ways we might theoretically demonstrate this. First, we might simulate by combining two (invented) variant callers WrongCaller (which always gets it wrong) and RightCaller (which only calls true positives), and show that minos basically outputs the same VCF as RightCaller. Alternatively, we could use empirical data from two variant callers with genuinely different strengths and weaknesses, and try to see if minos combines them to produce something better than either. The first option is of course very artificial, whereas the second option grapples with the reality that different callers focus on different sources of information (pileup, read-pairs, assembly etc) and so have different strengths (sensitivity/specificity and across the length/complexity spectrum of indels). We therefore chose option two.*

*Samtools and cortex have very different modes of operation and strengths - samtools uses pileups and paired-read information, and is relatively sensitive as a SNP caller, and is a pretty bad (error-prone) indel caller. Cortex uses de Bruijn assembly, and is extremely precise at calling both SNPs and indels, but has lower sensitivity. Combining the output of these two callers has the potential to be genuinely better than either, compensating for samtools awful indel-calling and cortex’s low SNP sensitivity and low power near repeated k-mers. This is indeed what we find. Supplementary Figure 3, reproduced here for simplicity, shows that the minos output has higher recall than either input caller. Precision is controlled, although not quite as good as Cortex.*

Would the results not be better if you picked three or more input methods?

*Reply: The improvement with each additional callset depends on how much extra value each new caller gives. A new caller needs to find true positive variants not found by previously incorporated callers in order to offer anything more. So the answer is - it depends. A natural followup question might then be - in that case, why not just use all available callers and combine them all? Would that maximise recall? The answer is yes, it would maximise recall, but it would also maximise CPU time.*

*In terms of the paper exposition:*

*1. We do not heavily emphasise this in the main text, but do mention that we use simulations first, to confirm that with simulated genomes and reads, minos (and the comparator tools) correctly combines callsets from cortex and samtools to select the true positive calls. The simulations were intended as a sanity check, designed to spot if there were strong effects for different sizes/types of indels or clustered variants.*

*2. We evaluate on a range of bacterial samples where we have high quality truth. These species cover a range of diversity, from a very low diversity species where one reference genome is relatively close to all samples (M. tuberculosis), through to a very high diversity recombining species where it is typically necessary to choose a reference genome within a clade of interest (K. pneumoniae), for which we test with multiple references.*

*To establish beyond doubt that minos does indeed support multiple callers we have now added the following:*

*1. We ran minos with samtools, cortex and a third tool, snippy (a wrapper for Freebayes). The results of this are shown in Supplementary Fig 4 and Supplementary Table 4 (both included below), showing negligible benefit of adding Snippy once Cortex and Samtools are already combined. Precision and recall are essentially unchanged for all 3 tools (Minos, GraphTyper, BayesTyper).*

*Supplementary figure 4: Effect of including variants from Snippy, in addition to Cortex and SAMtools, as input to BayesTyper, GraphTyper, and Minos. The change in precision and recall is shown across all simulated data and all bacteria data. Each point represents one run of one tool on one sample.*

*We have added this paragraph to the results section:*

*“We also investigated the effect of including more variant calls as input to BayesTyper, GraphTyper, and Minos. In principle a new caller adds value if it finds variants which are missed by the other callers. The above analysis was repeated, but with calls from snippy (https://github.com/tseemann/snippy) included together with those from SAMtools and Cortex. The results were almost identical: the mean precision and recall across all data for each tool was the same to two decimal places (Supplementary Table 4, Supplementary Figure 4), indicating that for these data, Snippy offers no additional benefit once Samtools and Cortex are combined.”*

*We have also updated our github documentation to clarify that we support any VCFs (not just SAMtools or cortex), saying that Minos requires “One or more VCF files of variant calls. The only requirement of these files is that they must contain the genotype field GT, and correspond to the reference FASTA file. All variants with a non-reference genotype call will be used (both alleles are considered for diploid calls).”*

*We also updated our paper to say in the first paragraph of the Results section: “It can accept VCF files from any source, using all records where the genotype (GT) field is present and has a non-reference call (records without this field are ignored).”*

According to the introduction, Minos addresses a problem with previous methods that they produce contradictory output. An example of the problem is discussed in Supplementary section 2 - where a deletion overlaps a SNP. A deletion call of "0" and SNP call of "1" is said to be contradictory/inconsistent, however I think this is a correct usage of the "*" allele in VCF. This output would mean that the SNP is present but the deletion is not. The format has each "variant event" (SNP/indel) in separate lines which is often preferred and is used by many other variant callers too, such as GATK and Octopus.

*Reply: Our reply comes in two parts. First, we respectfully suggest the text does not say quite what the reviewer thinks it says. The VCF spec allows a “*” genotype to mean this position is missing due to an overlapping deletion. So it would indeed not be legitimate for us to claim this was contradictory. However in the example we give, neither of the tools we discussed (BayesTyper and GraphTyper) actually called the * genotype. The * genotype is, in this example, a distraction. To save looking up, the two records are:*

NC_000962.3	397272 . TCGGCGCC	T

NC_000962.3	397275 . G	C,*

*The VCF contains a deletion, and then in the next record a SNP or *. Both tools called the reference allele in the first record, saying the deletion was NOT present, but the reference allele was present. This directly implies that at position 397275 the base should be G. But then, they call the C SNP allele (not the *) in the second record at that position 397275. These result in inconsistent records because they disagree about what base is present at the second site.*

*The second part of our reply is this: VCF format specification does indeed allow tool-developers to output multiple records which are related, such as a deletion in one record, which overlaps the next 200 SNP records. This is a good practical solution for tool developers, but it passes the problem forwards to the user as to how to parse these files. We do not address the bigger problem (overlapping long SVs and SNPs), but in minos we focus on SNPs and shorter indels, and work hard to combine overlapping records and thereby guarantee not to have conflicting records.*

The authors also provide a new tool for evaluating the output VCF files, Varifier. However, I found the motivation behind its need a bit unclear as there are already many good solutions available (RTG vcfeval, Illumina's Hap.py, syndip). What are the advantages of using Varifier over previous methods?

*Reply: We thank the reviewer for highlighting this limitation in our paper - we had indeed failed to motivate this decision. We have modified the second paragraph of the results, which introduces Varifier, to explain why we developed it instead of using existing tools. There are two key motivations.*

*First - in bacterial genomics it is now possible, using long reads, to generate a single contig whole genome assembly which is as close to “the truth” as we can currently reach. A truth assembly is unambiguous. If we want to assess precision and recall of calls in a VCF with respect to some reference, describing a sample S, the most direct approach is to check those calls against the truth assembly - does the truth contain the relevant bases at the position claimed in the VCF. Varifier is designed to do this very simply, by mapping any called variant, along with flanking sequence, to the truth assembly. If the mapping is unique, and the allele is in the right place with the right sequence, the truth is categorically established. If the mapping is unique, but the wrong allele is there, then this is a false positive. If the mapping is non-unique, varifier takes a conservative position, and says it cannot tell. We think this is the natural and safest approach when you have a haploid high quality truth.*

*Second - all other tools we are aware of (including those the reviewer mentioned - hap.py, RTG vcfeval, syndip) require a VCF of known truth variants as input. This makes sense in human/diploid genomics, where obtaining fully phased truth genomes is still a research question - an unphased VCF of truth calls is commonly the best you can do. However this approach is intrinsically problematic. Firstly, VCF is fundamentally ambiguous - there are multiple ways to represent a variant in VCF. As a result, there is no single truth VCF, at best you just have one possible truth VCF. We have added a real example from M. tuberculosis to the Supplementary text (section entitled “Example complex variant”), showing an insertion and SNP that are represented in different ways by different variant callers (including as two insertions and no SNP). Varifier can correctly take different VCF records that are consistent with the true sequence and confirm they are correct by mapping variant+flanks. In this example, passing two different VCF representations of the same sequence to hap.py leads to it (wrongly) thinking they are different. We expect other methods based on comparing VCF with a truth VCF would have the same problem. Where it is possible, validating directly against an assembly is fundamentally safer, in our opinion.*

*We include here a screenshot of the new supplementary section “Example complex variant”:*

*The new paragraph in our results section is:*

*“Existing tools to assess the accuracy of call sets, such as hap.py and RTG vcfeval, were developed for human diploid data and require truth variant calls in a VCF file. Such evaluations typically need to cope with uncertain phasing in the ``truth data''. However in our case, as is typical in bacterial genomics, the truth data is a polished (haploid) whole genome assembly assumed to contain no errors. We therefore developed a tool called Varifier to meet the need for a tool that uses such a truth sequence to determine the precision and recall of a call set. As described in full in the Methods section, Varifier evaluates each allele call by aligning the allele plus flanking sequence to the truth genome. This method is robust to complex variants, which can have more than one correct VCF representation. We found other tools could make errors around these types of variants -- an example is given in the Supplementary material. In this study, for each sample we used a truth genome assembled from long reads (PacBio or Oxford Nanopore), and polished using the same Illumina reads that were used for variant calling. We used Varifier and these truth genomes to benchmark Minos against GraphTyper and BayesTyper with simulated and real data.”*

I think there might be an error in Varifier's formula for calculating precision. For example, in the case of a simple false positive SNP (REF=A, ALT=T, TRUTH=A) then d(t,r)=0 and d(t,a)=1. This is supposed to get a score of 0 according to the text but with the given formula you get (0-1)/0 = -1/0

*Reply: Thank you for spotting this. It is an error in the description of the calculation in the manuscript. We actually force the score to be zero if d(t,r) = 0, and this is what the code does. We have amended the text to say this.*

Another concern with Varifier's precisions calculations is the use of edit distance since it means indels can weigh a lot more than SNPs. For example, a single 500 bp insertion false positive is equivalent to making 500 different SNP false positives. This means SNP precision has much lower effect on the final precision metric compared to other evaluation tools. Because of this, would you also consider stratifying somewhere the precision also by SNP and indel variants?

*Reply: We agree, and have clarified the description in the manuscript. A score of 1 is used for an insertion or deletion of any length, so that indels carry the same weight as SNPs. We did consider stratifying by SNPs and indels, however this is extremely difficult to do in a consistent manner because complex variants can be - and often are - called in multiple ways.*

Reviewer #2:

This paper describes a methodology implemented in a python software package called Minos. The goal of this method is to call variants or genotype individual samples w.r.t. reference cohort. The application focus is on bacterial genomes, where overlapping variants are more common.

The novelty of the method is to leave the discovery of variants to other established tools and focus on genotyping individual samples. The reads from bacterial species are fed through various variant calling programs and the variants recorded. Minos then takes the set of variants discovered and builds a variant graph from all programs and all samples. The reads for each samples are then aligned to the variant graph and the optimal variant is chosen as the output.

This approach, which the authors describe as "adjudicating" (I had too google that and double check the spelling), has the advantage of combining both output from several programs, each with their strengths and weaknesses, as well as sharing information across samples/species.

The introduction serves as a review of previous methods as well as motivating the problem. It is well written but I would urge the authors to make a clearer distinction between the problem of variant discovery (just finding the presence or absence of a variant) and genotyping (given the set of variants, which one does this individual have). Many programs treat this as a single problem, often termed as single sample variant calling in Human datasets, or separate them when doing joint variant calling.

*Reply: We thank the reviewer for highlighting this issue, and agree. We have added the following to conclude an early paragraph in the Introduction:*

*“In doing so we separate two processes which are typically bound together within a single variant-caller: the discovery of genetic variants, and the genotyping of these variants. In our schema, we allow different callers to do discovery, and then use our new method to adjudicate (i.e. genotype).”*

In particular, pg2, lines 59-page3 line3, this comparison of tools that cannot merge VCF files for bacterial species, it is mentioned that they were developed for human data primarily, but Graphtyper was also developed to go from BAM to genotypes for tens of thousands of human genomes and merging conflicting vcf's was not a primary design requirement. Overlapping variants are however dealt with in the variant discovery phase of the problem.

*Reply: We apologise for the text which the reviewer refers to; we now feel much of that paragraph was unnecessary or unhelpful, so we have replaced it with the following:*

*“Two other graph-mapping based tools are available: BayesTyper maps reads to a directed acyclic graph of informative kmers, and GraphTyper maps to local graphs of SNPs and indels from pre-mapped reads. BayesTyper is set up to take VCFs from different callers, combine them and genotype, much like Minos. GraphTyper's intended use is to genotype large human cohorts, either with SNP/indels it has discovered, or using a predefined VCF of structural variants. We compare Minos with both below.”*

Overall this is a well written paper that focuses on a problem of importance when genotyping bacterial samples.

*Reply: We thank the reviewer.*

The use of long read datasets for benchmarks as a gold standard is very important, but in the discussion the authors might want to address why we would want to use short read data at all.

*Reply: There are a couple of reasons for this. First, for any project where we want a consistent analysis done on a large cohort that is collected over several years (CRyPTIC started in 2015), nanopore data cannot be relied on to stay of the same quality across that timescale. In principle we could have kept the fast5 files and re-basecalled at the end, but again in 2015 this was not obviously true - they could easily have decided to break compatibility half-way through the project. Second, we really cared about indel calls and we had concerns about whether the quality of these could be trusted.*

*Moving forward, though, long read data is clearly going to be the standard for generating gold-quality benchmarks.*

Comments:

On pg 5, line 6, you run Graphtyper only as a genotyper and not as a variant discovery tool, I'm pleasantly surprised to see that it works on bacterial genomes, but it would be interesting to see how well it does when run as a variant discovery tool.

*Reply: In our benchmarks it doesn’t just work, it works very well. We agree that it would be interesting to look at its performance as a discovery tool, however that is outside the scope of our study, which is to adjudicate between sets of variant calls from arbitrary discovery tools.*

Pg 6, table 1

The comparison is quite close in many of the cases between the three methods. All of them are adjudicating the set of discovered variants and genotyping each sample, but it would be interesting to compare it to a) single sample variant callers and b) joint genotyping tools, I think the addition of those comparison would make it clear how important this step is.

*Reply: We thank the reviewer for bringing this up. We think this question combines a few issues, so we need to split them out.*

*1. Minos sets out to allow users to combine the output of different variant callers for a single sample, or to joint genotype a cohort. It is agnostic to the callers, and we are not “selling” one specific variant caller (or combination of callers). Our goal is to show it is possible to combine callsets and get something better. We do this through (simulation and) combining two variant callers with different strengths. In Supplementary Figure 3 we give the plots of precision and recall for Cortex, samtools, and their combination via minos showing minos with default filters pushes everything to the top right (higher precision and recall than either input).*

*2. When given a set of discovered variants, how does minos compare with other tools which can also do such genotyping? On our validation set all of the tools performed well, particularly graphtyper and minos. On the outbreak dataset, where we need to evaluate reference-call accuracy, not just non-reference calls, minos had significantly higher precision than the other tools. Finally, on the large cohorts, only minos was able to run. This last point is a reflection of tools being optimised for their intended application. The focus of minos is bacterial cohorts - had we applied to human data, then minos would probably have failed to scale acceptably (high RAM usage), whereas graphtyper and bayestyper would have run smoothly.*

*3. How does this fit into the bigger picture, where generally people focus either on single-sample variant callers, which combine discovery and genotyping, or joint genotyping methods? In terms of single-sample callers, the story is simple - minos provides a way to combine callers and improve results. Provided different callers have different strengths and detect slightly different SNPs/indels, it can be worthwhile to combine them.*

*In terms of joint genotyping: the community has already moved to doing something similar to minos for structural variants. There are multiple tools which take a list of SVs called by multiple different methods and then genotype a cohort using graphs (GraphTyper, paragraph, vg,..), so minos simply brings exactly that approach to SNPs and small indels. When the reviewer asks if we can compare with joint genotyping tools - that is exactly what we did with GraphTyper and BayesTyper. The GraphTyper2 paper and the BayesTyper papers precisely sell themselves as tools for (joint) genotyping cohorts with predefined SVs. The only difference for SNPs/indels is that the landscape is dominated by GATK, which only joint genotypes its own calls, and which does not yet fully support bacteria (there is something in development).*

*4. Finally, we consider the question of community need. There are a huge host of variant callers for SNPs/indels, all of which benchmark themselves and try to convince users that they are all anyone needs. However various large scale consortia, who care primarily about getting as close as possible to the truth, have realised that no caller is perfect, and that they need to combine results to improve them. The biggest example was the 1000 Genomes Project, which used a byzantine workflow that would be hard to reproduce (https://www.nature.com/articles/nature15393/figures/5) to combine SNPs from low-coverage population sequencing, SNPs from deep-sequencing at exons, and indels and structural variants from around 10 different variant-callers. More recently the Pan Cancer Analysis of Whole Genomes consortium decided that in order to obtain the highest possible quality variant calls, they needed to combine >5 different variant callers, using a combination of trusting things found by 2 callers (“consensus” approach), and a logistic regression model (SNV-MERGE) (*[*https://www.nature.com/articles/s41586-020-1969-6*](https://www.nature.com/articles/s41586-020-1969-6)*).*

*We would argue this is evidence that there is a clear need for rigorous methods for combining the output of different SNP and indel callers.*

Pg 7, line 49

The 23 and 26 hours on 2000 concurrent tasks is hard to compare, I would recommend reporting the total number of CPU hours.

*Reply: We have added this sentence to that paragraph: “The total CPU time was 787 core-days for the Mykrobe data set and 905 core-days for CRyPTIC.”*

pg 8, line 38. "which appear to cause rifampicin resistance"

How is this evaluated?

*Reply: We apologise for the ambiguous and unhelpful statement. We have corrected the text to read “Several rare indels present only in resistant samples were identified (red blocks in second panel of Fig 3)”.*

*We discuss the association of mutations in rpoB with rifampicin resistance at greater length below.*

pg 9, Figure 3.

Although it is cited that variations in the rpoB gene can cause rifampicin resistance, the only purpose of figure 3 is to show that the variants discovered with Minos are in many of the resistant species.

*Reply: We’d like to adjust that summary slightly. It is well known that mutations in rpoB cause rifampicin resistance, and that one region (the RRDR) is particularly enriched for such mutations. Some mutations are very common, and the association with resistance is strong, and in fact has been confirmed experimentally by allelic exchange, but others are sufficiently rare that it is not known. Nevertheless the WHO took a pragmatic decision and decided that for the purposes of public health, all non synonymous mutations and indels in the RRDR should be treated as resistance-causing, even the ones that have never been seen before. That’s the background. In that context, we show that minos is able to joint-genotype a large cohort of samples in this region, despite the host of overlapping SNPs and indels, providing clean data for the community to draw conclusions from. This is the first time this has been possible, as there has been no geno/phenotyping on a large enough scale to see all these rare SNPs and indels, and no method able to joint genotype at that scale. We give clear data for rare SNPs and indels for the first time, which can be followed up for example by allelic exchange experiments. We return to this below.*

Now there are two questions

1. How well did the other programs do in finding/calling these variants?

*Reply: We spent the early part of this paper showing that by combining callers, we improved recall and controlled precision. We used samtools and cortex as they have different strengths (SNPs and indels respectively, roughly speaking), and showed minos was better than either input. In this revision we show that adding Freebayes adds nothing over and above the Samtools and Cortex. That establishes minos/cortex/samtools as an improved method of calling over and above any of the individual callers. The key thing we do in this analysis is joint analyse the cohort despite multiple overlapping indels and SNPs. It also highlights several interesting non-synonymous SNPs which are never seen in resistant samples, despite being in the RRDR, contrary to the above-mentioned WHO pragmatic advice.*

2. If you compare other genes, not associated with rifampicin resistance, do you see the same enrichment of variants?

*Reply: In fact, no - we discovered this as we scaled-up joint-genotyping. The RRDR is one of a very few incredibly dense regions of variation in the genome, but there are a handful of others. The other major one is in katG, a gene associated with resistance to a different drug (isoniazid), where there are hundreds of bases where every single base is a quadrallelic SNP. However, we do not discuss this in the paper as it is outside our scope.*

3. How much information can be extracted from this, e.g. is there a statistical associatioon that these variants are more likely to be present in resistance and absent in non-resistant species? Any way you can quantify would be good.

*Reply: The causal relationship between common mutations in rpoB and resistance are well known, and have been experimentally validated. Further details can be found in this paper (https://pubmed.ncbi.nlm.nih.gov/24055765/) and for completeness sake we include their table showing which allelic exchange experiments have been done to confirm this, dating back as far as 1998:*

*Our study looks at a large cohort, covering rare and common mutations. Actually conducting association tests for specific mutations in this data is outside the scope of this paper, but is covered in other papers from our consortium preprinted at the same time. Earle et al conducted a GWAS (https://www.biorxiv.org/content/10.1101/2021.09.14.460272v1.full) and Carter at al specifically looked at rpoB mutations in the RRDR and looked at the different effect sizes of the different mutations (https://www.biorxiv.org/content/10.1101/2021.09.14.460353v2.full).*

pg 11, line 25

"combinations of alleles actually seen is used"

How often does this happen in the datasets?

*Reply: We have added this sentence to that paragraph: “This happened at 1,252 sites in the Mykrobe data set, covering 53,632bp of the reference genome, and at 1,195 sites (covering 50,324bp) in the CRyPTIC data set.”*

*We also fixed a typo: the cutoff is 500 alleles at a site, not 5000.*

*The full paragraph now reads:*

*“To process a large number of samples, heuristics are used to simplify the variant graph. First, the number of alleles is limited by only allowing deletions of length (by default) <=50bp. This prevents a combinatorial explosion where SNPs underneath the deletion can cause impractically large numbers of alleles: n biallelic SNPs generates 2^n alleles. Second, the number of alleles in a variant site is limited to (by default) 500. If generating all allele combinations at a site results in too many alleles, then only combinations of alleles actually seen in each sample are used. This happened at 1,252 sites in the Mykrobe data set, covering 53,632bp of the reference genome, and at 1,195 sites (covering 50,324bp) in the CRyPTIC data set.”*

pg 11, line 57

"mismatches between reads and graph are not allowed"

is this global or local, does the entire read have to match?

*Reply: The entire read must match, which is why we read-trim in advance. We have clarified the wording to say: “Note also that gramtools does exact matching of the full read length only - clipping the ends of the reads or mismatches between the read and graph are not allowed”.*

pg 12, line 8

"at a site", (missing A)

*Reply: Fixed.*

pg 13, line 46

"mappings that do not include the allele in the alignment"

This could be reworded to make it clearer, does this mean if the read is softclipped or something else?

*Reply: Apologies - you are right, this is about soft-clipping. We have added a clarification in brackets to the sentence: "mappings that do not include the allele in the alignment (ie if the start and end positions of the allele in the probe do not lie completely inside the start and end positions of the mapping because of soft-clipping) or have mapping quality equal to zero are ignored”.*

pg 14, line 12

1-\frac{d(t,a)}{d(t,r)}

would be clearer in my opinion

*Reply: We agree and have changed the manuscript to use this formula instead.*

Installation:

I tried to clone the repo and install, but I failed too build gramtools. When I did it manually (clone and run cmake) it complained that it required openMP, which my default mac compiler doesn't support.

*Reply: Thank you for attempting to install. Unfortunately we have not made support of Mac a priority, and focussed only on linux. We have added a Dockerfile to the Minos repository, to hopefully remove any compile/installation issues. We have also updated our release process so that singularity images are automatically added to the github release page.*

**Second round of review**

**Reviewer 1**

The authors have adequately addressed my comments.

I thank the authors for their revised version of the manuscript, and I would like to especially thank you for providing files showing the exact changes since the previous version.

**Reviewer 2**

The authors have address all of my concerns with the manuscript. I find that the manuscript has significantly improved with these revisions, especially the part on rifampicin which was harder for me to comprehend with limited knowledge about bacteria and drug resistance.
